# A comparative ultrastructure study of storage cells in the eutardigrade *Richtersius coronifer* in the hydrated state and after desiccation and heating stress

**DOI:** 10.1371/journal.pone.0201430

**Published:** 2018-08-10

**Authors:** Michaela Czerneková, Kamil Janelt, Sebastian Student, K. Ingemar Jönsson, Izabela Poprawa

**Affiliations:** 1 Department of Environmental Science and Bioscience, Kristianstad University, Kristianstad, Sweden; 2 Institute of Physiology, Academy of Sciences of the Czech Republic, Prague, Czech Republic; 3 Faculty of Medicine, Charles University, Prague, Czech Republic; 4 Department of Animal Histology and Embryology, University of Silesia in Katowice, Katowice, Poland; 5 Silesian University of Technology, Institute of Automatic Control, Gliwice, Poland; University of Colorado Boulder, UNITED STATES

## Abstract

Tardigrades represent an invertebrate phylum with no circulatory or respiratory system. Their body cavity is filled with free storage cells of the coelomocyte-type, which are responsible for important physiological functions. We report a study comparing the ultrastructure of storage cells in anhydrobiotic and hydrated specimens of the eutardigrade *Richtersius coronifer*. We also analysed the effect of temperature stress on storage cell structure. Firstly, we verified two types of ultrastructurally different storage cells, which differ in cellular organelle complexity, amount and content of reserve material and connection to oogenetic stage. Type I cells were found to differ ultrastructurally depending on the oogenetic stage of the animal. The main function of these cells is energy storage. Storage cells of Type I were also observed in the single male that was found among the analysed specimens. The second cell type, Type II, found only in females, represents young undifferentiated cells, possibly stem cells. The two types of cells also differ with respect to the presence of nucleolar vacuoles, which are related to oogenetic stages and to changes in nucleolic activity during oogenesis. Secondly, this study revealed that storage cells are not ultrastructurally affected by six months of desiccation or by heating following this desiccation period. However, heating of the desiccated animals (tuns) tended to reduce animal survival, indicating that long-term desiccation makes these animals more vulnerable to heat stress. We confirmed the degradative pathways during the rehydration process after desiccation and heat stress. Our study is the first to document two ultrastructurally different types of storage cells in tardigrades and reveals new perspectives for further studies of tardigrade storage cells.

## Introduction

Tardigrades represent an invertebrate phylum with many species that have evolved adaptations to survive extreme levels of dehydration and freezing [1, 2, 3, 4, 5, 6]. This has allowed them to inhabit some of the harshest environments on Earth (e.g., continental Antarctica), as well as equally extreme microhabitats in other areas (e.g., sun-exposed lichens and moss on rocks) [7, 8]. Tardigrades do not possess circulatory or respiratory systems, but their body cavity is filled with storage (or body cavity) cells, which float freely in the body cavity lymph [7, 8] or sometimes adhere to the basement membrane of other tissues [7]. These storage cells are responsible for important physiological functions, primarily nutrient transport and storage of mainly lipids but also polysaccharides and pigments such as carotenes [9, 10]. They also produce protein substances, which are gathered inside with lipid globules [10], and in some tardigrade species, vitellogenins are developed in the storage cells [11, 12]. Their energy storage function is well illustrated by the change in cell size over the oocyte maturation cycle, during which the cells grow in size from the early to the middle part of the cycle and decrease in size towards the end of the cycle as the energy demand of the developing eggs increases [11, 12, 13]. A similar pattern has been shown for the amount of energy reserve material in the cells [14]. Declines in storage cell size connected with a period of anhydrobiosis have been reported (*Richtersius coronifer* (Richters, 1903) [13]; *Milnesium tardigradum* (Doyère, 1840) [15]). However Czerneková and Jönsson [16] did not observe such changes after repeated periods of anhydrobiosis in *R*. *coronifer*.

Storage cells have also been used to study of DNA damage induced by desiccation. Neumann et al. [17] documented DNA fragmentation in storage cells of *M*. *tardigradum* after periods in the anhydrobiotic state and showed that fragmentation increased with time spent in the dry state (from 2 days to 10 months). Since many limnoterrestrial tardigrades are able to revive successfully after years of anhydrobiosis [18, 19] these animals seem to have an extraordinary capacity to repair the damage that arises and is accumulated during the dry state. However, the extent to which storage cells are damaged ultrastructurally after long-term anhydrobiosis or exposure to other stressors remains to be documented.

High temperature is an agent that may disrupt cell structures such as membranes, DNA and proteins. Relatively few studies have evaluated thermotolerance in tardigrades. In the hydrated state an upper tolerance level of 36°C and 38°C after 24 h exposure was reported in *Borealibius zetlandicus* (Murray, 1907) [20] and in *Macrobiotus harmsworthi* (Murray, 1907), respectively [21]. In the anhydrobiotic state short-term (1 h) heat tolerance is considerably higher, and tolerances up to approximately 100°C have been reported [22], but variations in tolerance among tardigrade species are considerable [22, 23]. Older studies have reported even higher tolerances (up to 151°C for 30 min. exposure [24]). In *R*. *coronifer*, the tardigrade used in the present study, 1 h exposure of temperatures up to 70°C did not affect survival, but at 80°C, survival was below 20%, and at 85°C, it was near zero [25]. Most studies on heat tolerance in desiccated tardigrades have used short exposure times (1 h), but Rebecchi et al. [26] exposed anhydrobiotic tardigrades of the species *Paramacrobiotus richtersi* (Murray, 1911) to 37°C at 30–40% RH for up to 21 days, with no effect on survival. However, a separate experiment showed that the survival of dry animals over a 21 day period was inversely related to the relative humidity at which the animals were kept [26]. There were also indications of DNA damage (single-strand breaks) in animals exposed to the highest relative humidities. Analyses of how exposure to heat affects the cell ultrastructure of tardigrades have not been reported.

In this study, we compared the ultrastructure of storage cells in active and anhydrobiotic specimens of the eutardigrade *R*. *coronifer*. We also examined if storage cell structure was affected by heat stress.

## Materials and methods

**Fig 1:**
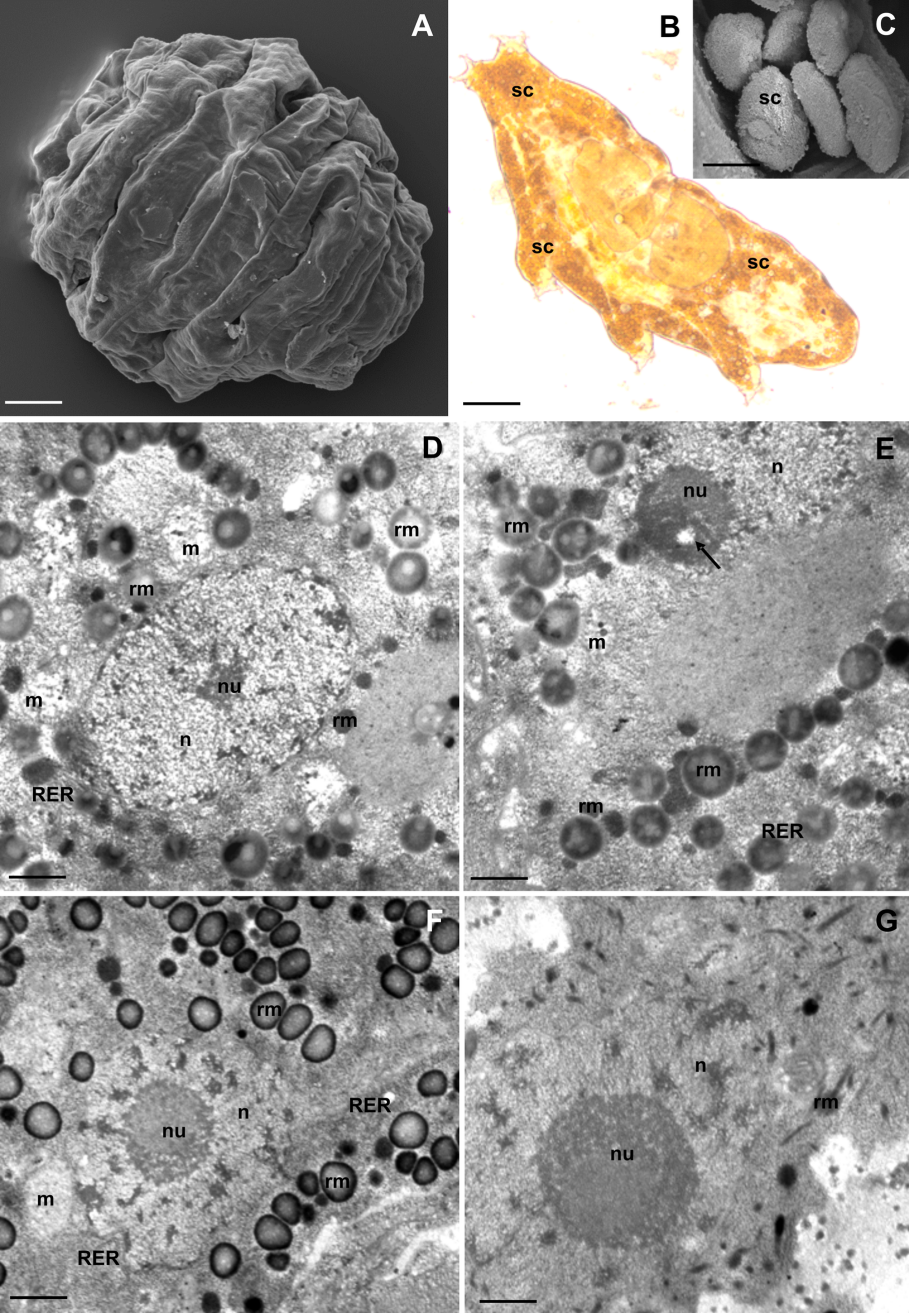
Storage cells (SC) of *R*. *coronifer*. **(A)** Tun, SEM. Bar = 30 μm. **(B)** Active animal, LM. Bar = 20 μm. **(C)** Storage cells, SEM. Bar = 4 μm. **(D-G)** Ultrastructure of SC of non-experimental specimens, TEM: nucleus (n), nucleolus (nu), mitochondria (m), rough endoplasmic reticulum (RER), spheres of reserve material (rm). **(D-E).** SC of male specimens. **(D)** Bar = 0.58 μm. **(E)** Bar = 0.5 μm. **(F-G)** SC of female specimens. **(F)** SC of the first type during vitellogenesis, nucleolus vacuole (arrow). Bar = 0.8 μm. **(G)** SC of the second type. Bar = 0.5 μm.

We used the eutardigrade *R*. *coronifer* (Fig 1A and 1B), a species belonging to the order Parachela, family Macrobiotidae. This species has well-documented anhydrobiotic ability (e.g., [23, 27, 28, 29]). The specimens were obtained from mosses at the Alvar habitat of the Swedish Baltic Sea island Öland [30]. Previous studies have shown that the population consists almost exclusively of females [30]. More than one tardigrade extraction method was used. Tardigrades were extracted from the sample by soaking dry mosses for 2 up to 4 h in distilled water, followed by mixing and shaking them off. The sediment/water mixture containing tardigrades was poured into cylinders and put aside for half an hour for decantation [31], additionally tardigrades were extracted with sieves (mesh size 250 and 40 μm) under running tap water. Only medium–large size (ca. 0.5–1.0 mm body length) specimens were used. Specimens analysed in the tun stage were desiccated individually on filter paper under 95% relative humidity (RH) using a saturated salt solution (KNO_3_) in a closed container at room temperature (see, e.g., [32]). In specimens analysed in the hydrated state, the stage of oogenesis (see, e.g., [12]) was recorded in order to evaluate if storage cell structure differed between oogenesis stages.

### I. Non-experimental analyses of storage cells in desiccated and hydrated specimens

#### Light and transmission electron microscopy

Forty-five active animals and fifteen tuns were fixed with 2.5% glutaraldehyde in a 0.1 M sodium phosphate buffer (pH 7.4, 4°C, 2 h). The material was post-fixed with 2% osmium tetroxide in a 0.1 M phosphate buffer (4°C, 2 h) and washed in a 0.1 M phosphate buffer. After dehydration in increasing concentrations of ethanol (30, 50, 70, 90, 95 and 100%, each for 15 min), a mixture of 100% ethanol and acetone (1:1, 15 min), and acetone (2 x 15 min), the material was embedded in epoxy resin (Epoxy Embedding Medium Kit; Sigma). Semi- (800 nm thick) and ultra-thin (50 nm thick) sections were cut on a Leica Ultracut UCT25 ultramicrotome. Semi-thin sections were stained with 1% methylene blue in 0.5% borax and observed with an Olympus BX60 light microscope. Some of the semi-thin sections (without staining with 1% methylene blue in 0.5% borax) were used for the histochemical methods (see below). Ultra-thin sections were put on formvar-covered copper grids and stained with uranyl acetate and lead citrate. The material was analysed with a Hitachi H500 transmission electron microscope at 75 kV.

Additionally, ultrathin sections from ten hydrated and five desiccated specimens of *R*. *coronifer* were used in order to evaluate the presence of structurally different storage cells. In each section, 100 randomly selected cells were analysed.

Ultrathin sections of the *R*. *coronifer* bodies (five active animals and five tuns) were also used to estimate the diameters of storage cells in active animal and in tun. Fifty storage cells in each of five active animals and fifty storage cells in each of five tuns were measured. The active animals and the tuns were at the same stage of oogenesis (late vitellogenesis).

#### Scanning electron microscopy

Five active animals and five tuns were fixed in 10% ethanol (2 min) and dehydrated in a graded concentration series of ethanol (20, 30, 40, 50, 60, 70, 80, 90, 4 x 100% each for 2 min), followed by a hexamethyldisilazane (HMDS) chemical drying series (ethanol:HMDS at 2:1, 1:1, 1:2 each for 10 min) and 100% HMDS (then allowed to air dry). Dried specimens were mounted on SEM stubs and coated with gold in a Pelco SC-6 duster. The material was examined using a Hitachi UHR FE-SEM SU 8010 scanning electron microscope.

#### Histochemistry and immunohistochemistry

Detection of polysaccharides (PAS method). Semi-thin sections (from 5 active specimens and 3 tuns) were treated with 2% periodic acid (10 min, room temperature) in order to remove the osmium tetroxide from the tissue, stained with Schiff’s reagent for 24 h at 37°C [33] (Litwin, 1985), washed in tap water (15 min) and observed with an Olympus BX60 light microscope.

Detection of proteins (Bonhag’s method). Semi-thin sections (from 5 active specimens and 3 tuns) were treated with a 2% solution of periodic acid as in the PAS method, stained with bromophenol blue (BPB) (24 h at 37°C) [33] (Litwin, 1985), washed in tap water (15 min) and observed with an Olympus BX60 light microscope.

Detection of lipids. To detect lipids, semi-thin sections (from 5 active specimens and 3 tuns) were stained with Sudan black B [33] at room temperature for 20 min, washed quickly in 50% ethanol then in distilled water and observed with an Olympus BX60 light microscope.

BODIPY 493/503 –detection of lipids. Ten hydrated specimens and five tuns of *R*. *coronifer* were punctured with a thin wolfram needle for better penetration of reagents inside the body and fixed with 2.5% paraformaldehyde in TBS (45 min, room temperature). The specimens were then washed in TBS and stained with 20 μg/ml BODIPY 493/503 (Molecular Probes) (30 min in darkness/room temperature). The material was then washed in TBS, stained with Hoechst 33342 (1 μg/ml, 20 min, room temperature), washed in TBS and whole-mounted on microscopic slides. The material was analysed with an Olympus FluoView FV 1000 confocal microscope. Excitation at 493 nm was provided by a multi-line argon laser.

Immunolabelling with anti-phosphohistone H3—a mitotic-specific antibody (for detection of cell proliferation). Ten hydrated specimens of *R*. *coronifer* were punctured with a thin wolfram needle for better penetration of the chemical reagents. The material was washed with TBS (5 min), 0.1% Triton X-100 in TBS (5 min) and incubated in 1% BSA in TBS (1 h, room temperature) without fixation. The material was then incubated overnight (16 h) in a 1:100 dilution of anti-phosphohistone H3 antibodies (Millipore) in 1% BSA in TBS. After incubation, the specimens were washed twice with TBS (5 min) and then incubated in a 1:200 dilution of goat anti-rabbit IgG Alexa-Fluor 488 conjugated secondary antibody diluted in 1% BSA in TBS (2 h, room temperature in darkness). Afterwards, the specimens were stained with DAPI (1 mg/ml, 20 min, room temperature in darkness). The material was mounted onto slides and analysed with an Olympus FluoView FV1000 confocal microscope. Excitation at 488 nm was provided by an argon/krypton laser.

TUNEL assay (detection of cell death). Ten hydrated specimens of *R*. *coronifer* were punctured with a thin wolfram needle, incubated in a permeabilization solution (0.1% sodium citrate) (2 min on ice in 4°C) and washed in TBS (3×5 min). The specimens were then stained with a terminal deoxynucleotidyl transferase dUTP nick end labelling (TUNEL) reaction mixture (In Situ Cell Death Detection Kit, TMR red, Roche; 60 min at 37°C in the dark). A negative control was prepared according to the labelling protocol. The material was analysed with an Olympus FluoView FV 1000 confocal microscope. Excitation at 594 nm was provided by a multi-line argon laser.

### II. Effects of long-term desiccation and heating on storage cell structures

#### Experimental design

We evaluated ultrastructural changes in storage cells after (i) desiccation of tardigrade specimens for six months and (ii) desiccation of tardigrade specimens for six months + heating at 50°C for 24 h. For both groups, analyses of storage cells were performed both before (i.e., still desiccated specimens) and after rehydration (three and five hours post-rehydration). Three specimens in each of the four categories (heated desiccated, non-heated desiccated, heated rehydrated, and non-heated rehydrated) were used. In addition, 14 specimens each from category (i) and (ii) were prepared for analysis of survival. To analyse storage cell ultrastructure, we used transmission electron microscope and histochemical methods for detection of lipid, proteins and polysaccharides.

#### Anhydrobiotic induction, heating and rehydration

Extracted animals were washed thoroughly with distilled water to remove adherent particles. Five hours later the hydrated specimens were dehydrated individually on small squares (5 cm^2^) of filter paper at 95% relative humidity (RH) using a saturated salt solution (KNO_3_) in a closed container at room temperature. After 24 h, the filter papers with dehydrated specimens were enclosed in small plastic bags and kept in the laboratory (room temperature) for 6 months. Immediately after the 6 month period, specimens in the heating group (animals determined for heating in incubator) were heated in an incubator at 50°C for 24 h. Half of them (n = 14) were then fixed in the desiccated state and prepared for microscopy, and the other half (n = 14) were rehydrated individually in circa 4 ml of distilled water in Petri dishes (60 x 15 mm). The same procedure, except of heating, was used for the non-heated specimens. Specimens used for post-rehydration analyses were rehydrated individually in Petri dishes (60 x 15 cm) with distilled water for 3 or 5 h before fixation for ultrastructure analysis. Specimens used for survival analysis were checked after 3 and 5 h post-rehydration. Animals were recorded as alive if they were active (slowly moving and fully moving or fully active) and were still moving after 2 more hours (5h and 7h).

#### Light and electron microscopy

Ten desiccated (6 from the experimental and 4 from the control group) and ten rehydrated (6 from the experimental and 4 from the control group) specimens were prepared for analysis with a transmission electron microscope (Hitachi H500 at 75 kV) as described earlier (see I. Non-experimental analyses of storage cells in desiccated and hydrated specimens, light and transmission electron microscopy).

#### Histochemical analysis

Detection of polysaccharides (PAS method). Semi-thin sections (from 4 active specimens and 3 tuns) were used for detection of polysaccharides. The same method as in the non-experimental study was used; see the description above.

Detection of proteins (Bonhag´s method). Semi-thin sections (from 4 active specimens and 3 tuns) were used for detection of proteins. The method was described earlier (see the non-experimental study, the description above).

Detection of lipids. Semi-thin sections (from 4 active specimens and 3 tuns) were used for detection of lipids. The same method as in the non-experimental study was used; see the description above.

Ethics statement: The study did not involve endangered or protected species, and moss samples were not collected within an area where permission was required.

## Results

### Non-experimental analyses of storage cells

#### Storage cells of hydrated specimens

The body cavity of *R*. *coronifer* was filled with fluid and storage cells (Fig 1B). The cells of examined specimens had ameboidal or spherical shapes (Fig 1B and 1C). The average diameter of cells in the five specimens examined for cell size was 15.36 μm (S1 Table, S1 File). Among all analysed specimens (eighty active), we found only one male. All desiccated animals (twenty-five tuns) were females.

#### Storage cells of the male

Only one type of storage cells (Type I) was observed in the male. These cells had an ameboidal shape. The large nucleus (Fig 1D and 1E) with a non-homogenous nucleolus was located in the centre of each cell (Fig 1E). The nucleolus was composed of two types of material with different electron density. A small nucleolus vacuole with low electron density was observed in the nucleolus (Fig 1E). The cytoplasm was filled with organelles, such as ribosomes, mitochondria and short cisterns of rough endoplasmic reticulum (Fig 1D and 1E). Moreover, non-homogenous spheres of different size and electron density were observed in the cytoplasm (Fig 1D and 1E). Most of the electron-dense spheres were filled with granules of lower electron density (Fig 1D and 1E). Medium electron-dense spheres and spheres of high electron density were also distinguished in the cytoplasm of the storage cells (Fig 1D and 1E).

#### Storage cells of females

Two types of storage cells were found in females. The cells of the first type (Fig 1F) were similar to those observed in the male, thus of Type I. Their ultrastructure differed in relation to the stages of oogenesis (see below). The cells of the second type (Type II) had an ameboidal shape (Fig 1G), and their ultrastructure was similar during all stages of oogenesis. The centre of each cell of Type II was occupied by a large lobular nucleus with a large non-homogenous nucleolus. The external part of the nucleolus had a higher electron density than its internal part (Fig 1G). The cytoplasm of these cells was poor in organelles. It contained ribosomes, mitochondria, a few short cisterns of rough endoplasmic reticulum and several small electron-dense granules. Among the observed storage cells, we found on average 7.2% cells of the Type II.

#### Ultrastructural differences in storage cells of Type I in relation to stage of oogenesis

**Fig 2:**
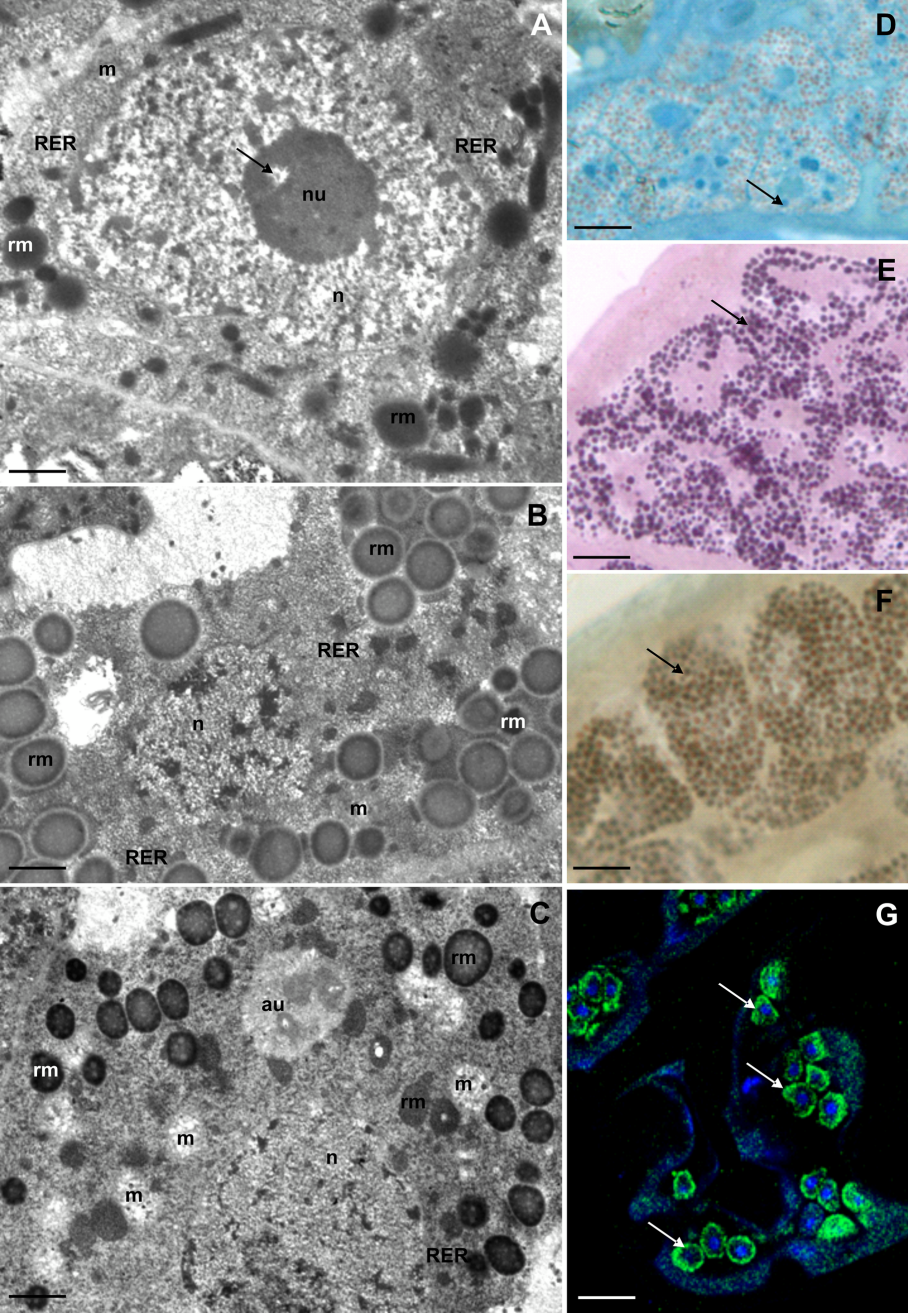
Ultrastructure and histochemistry of the SC of the first type during different stages of oogenesis. **(A-C)** Ultrastructure of SC, TEM: nucleus (n), nucleolus (nu), mitochondria (m), rough endoplasmic reticulum (RER), spheres of reserve material (rm). **(A)** Previtellogenesis, nucleolus vacuole (arrow). Bar = 0.47 μm. **(B)** Late vitellogenesis. Bar = 0.57 μm. **(C)** Late choriogenesis, autophagosome (au). Bar = 0.65 μm. **(D-G).** Histochemical staining of SC, arrow indicates positive reaction: **(D)** BPB staining, LM. Bar = 4 μm. **(E)** PAS method, LM. Bar = 3.5 μm. **(F)** Sudan Black B staining, LM. Bar = 3 μm. **(G)** BODIPY 493/503 and DAPI staining, confocal microscopy. Bar = 10 μm.

The process of tardigrade oogenesis can be divided into three major stages: previtellogenesis (organelle accumulation and mRNA synthesis), vitellogenesis (early, middle and late vitellogenesis—yolk synthesis and accumulation) and choriogenesis (egg shells formation) [34, 35, 36, 37]. To see if storage cell structure differed between oogenesis stages, we analysed 10 specimens in previtellogenesis, 24 specimens in vitellogenesis, and 10 specimens in choriogenesis. During previtellogenesis, the central part of each storage cell was occupied by a large nucleus with a large non-homogenous nucleolus (Fig 2A). The internal part of the nucleolus had a lower electron density than its external part. Moreover, a small nucleolus vacuole with a low electron density was present (Fig 2A). At this stage the cytoplasm was filled with ribosomes, short cisterns of rough endoplasmic reticulum, few mitochondria and a small amount of reserve material (Fig 2A). The reserve material had the form of smaller and larger spheres of different electron density. Smaller spheres were electron-dense, while the larger spheres had lower electron density (Fig 2A).

Subsequently, during vitellogenesis, an increase in the number of mitochondria and spheres of the reserve material were observed in the cytoplasm of the storage cells (Fig 1F). The central part of each cell was still occupied by the large nucleus with a large non-homogenous nucleolus. However, the nucleolus vacuole was not observed at this stage (Fig 1F). The stored spheres of the reserve material had different sizes and electron density. Most of the spheres had medium electron density. They possessed a high electron-dense external ring and granules of lower electron density. Moreover, smaller homogenous electron-dense and medium electron-dense spheres were observed (Fig 1F).

During late vitellogenesis and the beginning of choriogenesis the number of mitochondria, cisterns of rough endoplasmic reticulum, and the amount and type of reserve material accumulated in the cytoplasm of the storage cells did not change with respect to the stage of vitellogenesis (Fig 2B). The amount of reserve material decreased significantly at the end of choriogenesis (Fig 2C). Moreover, the number of mitochondria increased at this time. Additionally, some autophagosomes with fibrous medium electron dense material inside them were observed in the cytoplasm (Fig 2C). The amount of reserve material decreased until the end of oviposition. A very small amount of proteins (Fig 2D) and large amounts of polysaccharides (Fig 2E) and lipids (Fig 2F and 2G) were accumulated in the cytoplasm of the storage cells of the analysed species.

**Fig 3:**
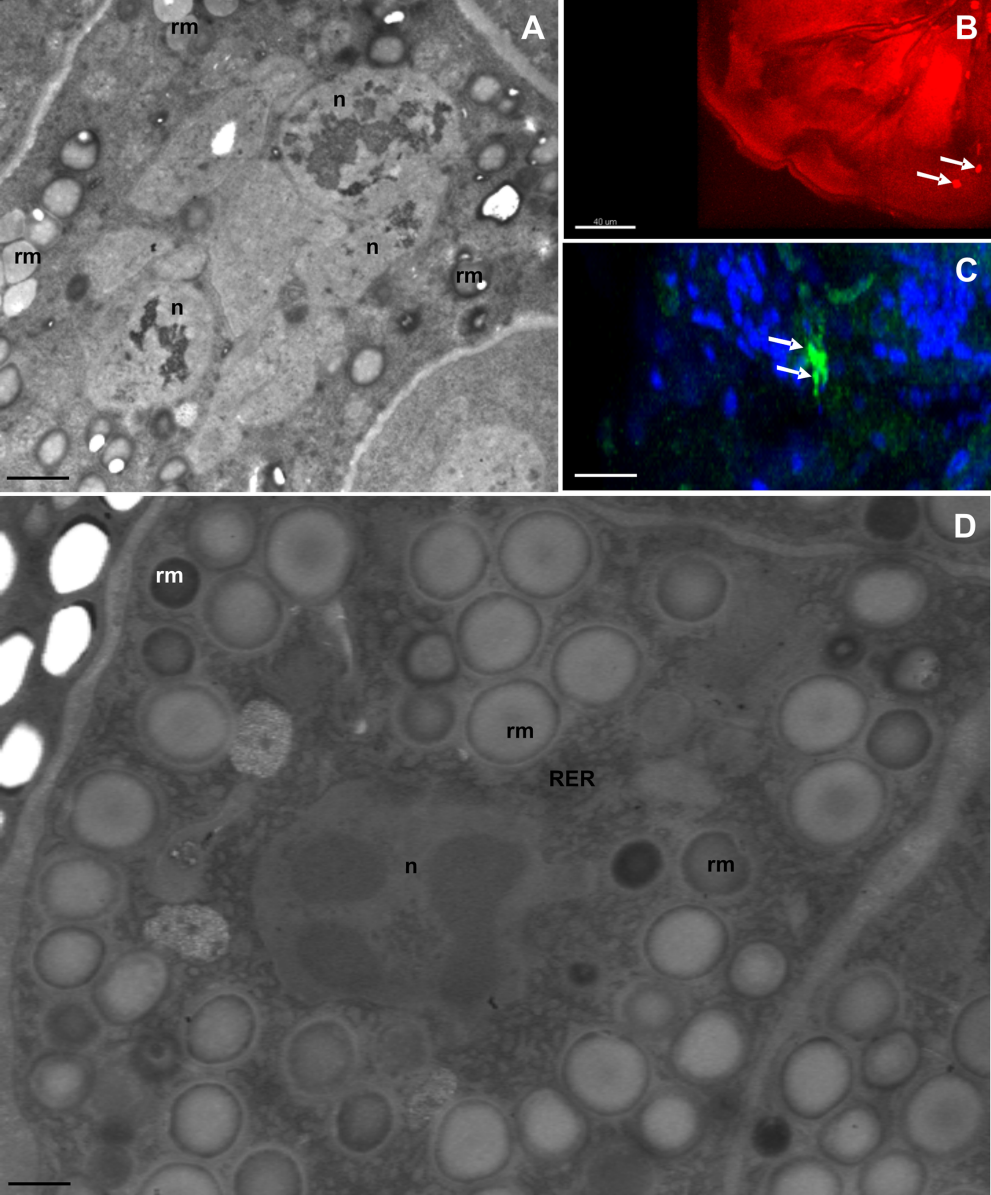
Ultrastructure of storage cells (SC) of desiccated specimens. **(A)** Degeneration of SC, nucleus (n), spheres of reserve material (rm), TEM. Bar = 0.6 μm. **(B)** Detection of the cell death, arrow indicates nucleus of the apoptotic cell, TUNEL, confocal microscopy. Bar = 40 μm. **(C)** Detection of the cell proliferation, arrow indicates nucleus of the proliferating cell, anti-phosphohistone H3 staining, confocal microscopy. Bar = 25 μm. **(D)** Ultrastructure of SC of desiccated specimen: nucleus (n), mitochondria (m), rough endoplasmic reticulum (RER), spheres of reserve material (rm), TEM. Bar = 0.36 μm.

We observed indications (not quantified) of degeneration of some individual storage cells. The cytoplasm of these cells was electron-dense; many clusters of heterochromatin occurred in the neighbourhood of their nuclear envelope, and their nuclei underwent fragmentation (Fig 3A). The fragmentation of DNA in nuclei (Fig 3B) indicates an apoptotic cell death of these cells.

Sporadically, divisions of the storage cells were observed (Fig 3C). Since we did not obtain images of the dividing cells by transmission electron microscopy, it was not possible to determine if dividing storage cells belonged to the first or second type.

#### Storage cells in desiccated specimens

We analysed storage cell ultrastructure in fifteen tuns during different stages of oogenesis. The storage cells of tuns were shrunken and had an ameboidal shape (Fig 3D). The average diameter of the cells in the five tuns examined for cell size was 11.8 μm (S1 Table), which is significantly smaller than cells of hydrated specimens (Mann-Whitney U-test, U = 0.0, P = 0.005, N = 10). The general characteristics of desiccated storage cells of *R*. *coronifer* were reported in our previous article [38].

#### Immunolabelling

We detected cell divisions in 2 specimens in late stage of oogenesis with the use of immunolabelling with anti-phosphohistone H3, a mitotic-specific antibody (for detection of cell proliferation). In the first and second specimens, 7 nuclei and 5 nuclei, respectively, were found in a mitotic stage. In one specimen in a late oogenesis stage, 4 nuclei were detected with TUNEL labelling for detection of cell death.

### Experimental study on long-term desiccation and heating

#### Survival of specimens

The survival of specimens desiccated (but not heated) for six months was 100% (n = 14). All of the non-heated specimens (n = 14) were fully active (coordinated body movements, directional movements forwards as well as to the side angles, using all legs, moulting of cuticle) within 3 h after rehydration. The survival of heated specimens was 40% (6 survivals, n = 14). Among the heated survivors, 50% were fully active after 3 h of rehydration, whereas the other specimens showed only some slow moves in some legs, and required 5 h of rehydration to resume full activity.

#### Storage cell ultrastructure of heated and non-heated specimens

**Fig 4:**
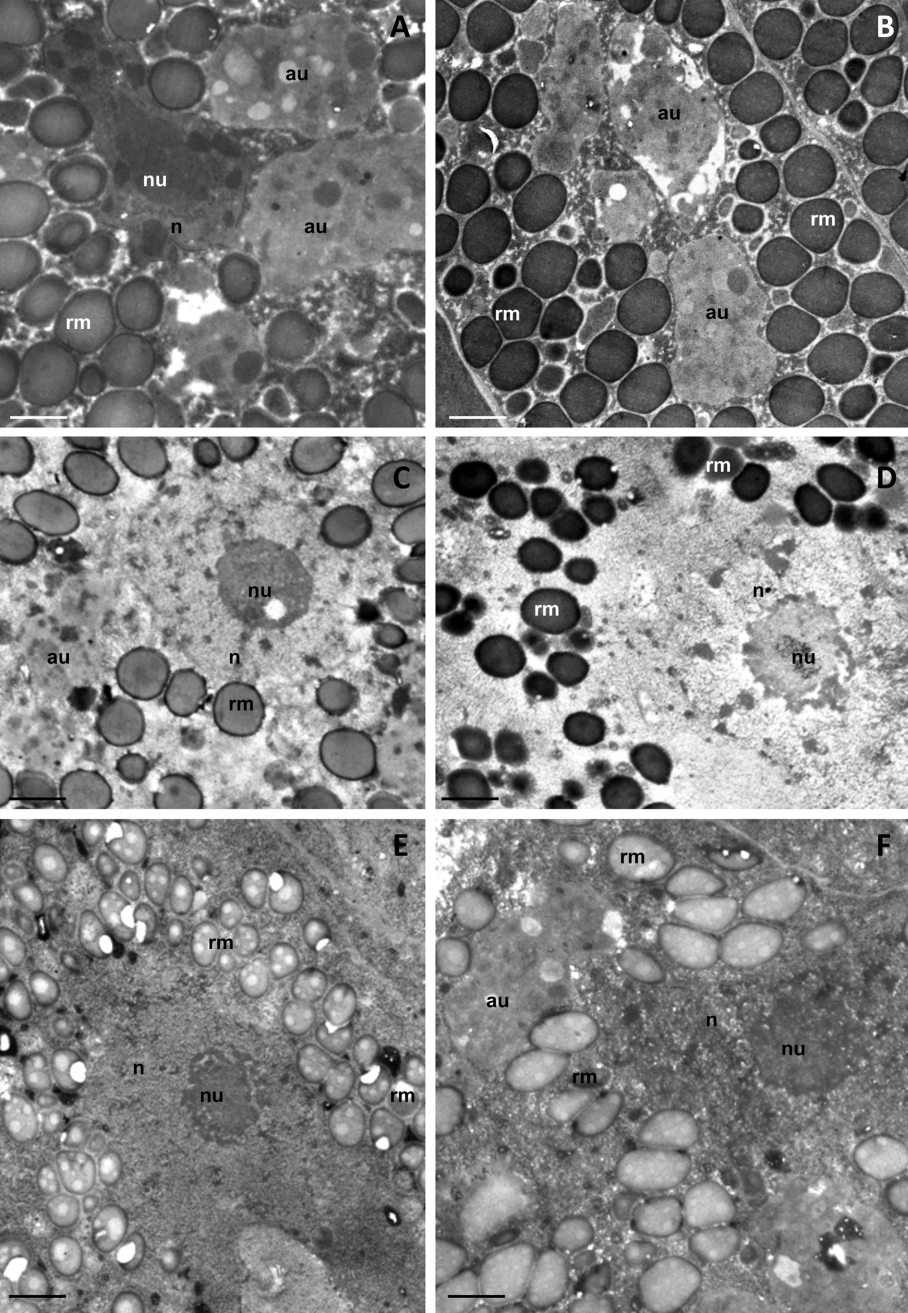
Ultrastructure of storage cells (SC) of experimental specimens. Autophagosome (au), nucleus (n), nucleolus (nu), spheres of reserve material (rm), TEM. **(A)** SC of non-heated 6 month old desiccated specimens. Bar = 0.65 μm. **(B)** SC of heated 6 month old desiccated specimens. Bar = 0.65 μm. **(C)** SC of non-heated 3h rehydrated specimens. Bar = 0.8 μm. **(D)** SC of 3h rehydrated specimens, which were heated prior rehydration. Bar = 0.8 μm. **(E)** SC of non-heated 5h rehydrated specimens. Bar = 0.95 μm. **(F)** SC of 5h rehydrated specimens, which were heated prior rehydration. Bar = 0.8 μm.

The storage cell ultrastructure of heated and non-heated desiccated specimens appeared similar (Fig 4A and 4B). The cells were shrunken with an amoeboid shape, and the cytoplasm was electron dense and entirely filled with membrane coated spheres (Fig 4A and 4B). The centre of all observed cells was occupied by an irregular nucleus with a distinct nucleolus and dense heterochromatin masses (Fig 4A). Large autophagosomes were present in the cytoplasm of the storage cells of both heated and non-heated specimens (Fig 4A and 4B). Differences between cells were only found in the density of spheres. In the heated specimens, the non-homogenous larger spheres were filled with granules of lower electron density, while the spheres of non-heated specimens were homogenous (Fig 4A and 4B).

All rehydrated cells had a circular or amoeboid shape, and there was no apparent difference in ultrastructure between non-heated and heated specimens. After 3 h of rehydration, the cytoplasm was electron lucent and containing a circular nucleus with a distinct nucleolus (Fig 4C and 4D). The cytoplasm of both heated and non-heated specimens contained non-homogenous and homogenous membrane coated spheres of various electron densities (Fig 4C and 4D). Autophagosomes were observed in the cell cytoplasm (Fig 4C). After 5 h of rehydration, the storage cells contained nuclei with a distinct nucleolus in both non-heated and heated specimens (Fig 4E and 4F). The nuclei of some cells were fragmented and degraded (not shown). The spheres with reserve material, filling the cytoplasm of 5 h rehydrated cells, were non-homogenous and contained electron lucent and medium electron dense bodies (Fig 4E and 4F). Large autophagosomes were also observed in the cytoplasm after 5 h rehydration (Fig 4F).

Large amounts of lipid and polysaccharides and a low amount of protein were detected in the storage cells of all examined specimens (not shown).

## Discussion

### Storage cells in active and anhydrobiotic animals

We compared storage cells of active and anhydrobiotic specimens of *R*. *coronifer* in all oogenetic stages. We observed dividing as well as apoptotic storage cells in active animals. Some differences between storage cells of active and desiccated specimens of *R*. *coronifer* were observed. During desiccation, the storage cells slightly changed their shape as the water evaporated, and a low water content and condensed cytoplasm resulted in a higher electron density of condensed cytoplasm and nucleoplasmic matrix [38], which confirmed the observations of Walz [39]. The storage cells of the desiccated specimen also had significantly smaller cells than the active animals. In cells of active specimens, we observed a higher number of autophagosomes at the end of choriogenesis and after 3–5 h of rehydration.

Autophagic pathways allow cells to eliminate large portions of the cytoplasm, aberrant protein aggregates, damaged organelles or invading bacteria. Structures targeted for degradation are gradually surrounded with the phagophore, and double membrane vesicles called autophagosomes are formed [40]. Since autophagy is known to be a major factor in the turnover of long lived proteins, the presence of autophagosomes indicates degradative pathways during dehydration and rehydration processes in cells as a response to damage and/or starvation. Autophagy might therefore be more common in cells that have undergone dehydration than in the cells of healthy, well fed animals [40]. In tardigrades, autophagy was also observed in the digestive cells of the midgut epithelium, and in trophocytes, at the end of oogenesis [36, 40, 41]. In case of the midgut epithelium, initially, when the stressor (infection by pathogens, starvation) was weak, autophagy was activated. However, when the stressor was too strong, autophagy initiated necrosis [36, 41]. In trophocytes, autophagy is the first step of cell degeneration, which is followed by apoptosis [40].

We verified ultrastructurally two types of storage cells, which differed in cellular organelle complexity, amount and content of reserve material and connection with oogenetic stages. The Type I occurred in both the male and females, while Type II was found only in females. One of the features of Type I storage cells was the presence of nucleolar vacuoles. Nucleolar vacuoles, also called nucleolar cavities or interstices, are rather characteristic of plant cells, are rarely visible in animal nucleoli, and represent high nucleoli activity (RNA synthesis) [42, 43, 44]. In plant cells, they are possibly connected with mitosis, particularly in condensation and decondensation of chromosomes [45]. In females of *R*. *coronifer*, the nucleolus vacuoles were ultrastructurally related to oogenetic stages with respect to the presence/absence of this structure and the amount and type of reserve material. These cells were in general filled with plenty of mitochondria, cisterns of rough endoplasmic reticulum and specific spheres of different electron densities, particularly lipid reserve material. We therefore assume that storage cells of Type I have intense metabolic activity and that their main function is storage and distribution of energy [13, 16]. This is in line with previous studies on storage cells in other tardigrade species, indicating intense metabolic activity [8, 11, 12, 14, 46]. In relation to overall organelle complexity differences and oogenesis, it seems that the function of nucleolar vacuoles in tardigrades is related to changes in nucleolic activity of storage cells during different stages of oogenesis, which was previously suggested in other organisms [12, 42, 43, 44]. Moreover, the nucleolar vacuole serves as a diagnostic feature in some species, e.g., Caryophillidea (Cestoda). Nucleolar vacuoles were also observed in storage cells of *Hypsibius exemplaris* Gąsiorek, Stec, Morek and Michalczyk, 2018, *Macrobiotus polonicus* Pilato, Kaczmarek, Michalczyk and Lisi, 2003 and *Xerobiotus pseudohufelandi* (Iharos, 1966) [14]. Nevertheless, their specific function in tardigrades cells (similar to other animal cells) is still unknown.

In tardigrades, yolk material accumulated in the cytoplasm of the oocytes is synthesized by the oocyte and their sister cells (trophocytes); however, sometimes the yolk precursors are synthesized by storage cells or the cells of the midgut epithelium [11, 12, 36]. The synthesis of yolk precursors by storage cells was reported in some Macrobiotidae species, e.g., *Dactylobiotus dispar* (Murray, 1907) [12], *M*. *polonicus* and *Paramacrobiotus richtersi* (Murray, 1907) [11], as well as in some other species, e.g., *Hypsibius exemplaris* and *Isohypsibius granulifer granulifer* (Thulin, 1928) [14]. In some tardigrades, the amounts of reserve material accumulated in the storage cells increases gradually during previtellogenesis and start to decrease during vitellogenesis and choriogenesis [11, 12, 14]. These observations indicate participation of the storage cells in yolk precursor synthesis. We observed that the fine ultrastructure of the first storage cell type is in general similar to other Parachela species [14] but differs in stored reserve material. During yolk synthesis (vitellogenesis), the amount of reserve material in storage cells of *R*. *coronifer* increases, but no changes were observed during late vitellogenesis and choriogenesis. Late vitellogenesis occurs at the simplex stage, a start of moulting stage, when the bucco-pharyngeal apparatus is absent or incomplete, while the late choriogenesis is connected with the moulting process [14, 47, 48]. At these stages, the animals do not eat, and the ovaries are large and oppress the midgut lumen. Since these storage cells during late vitellogenesis/choriogenesis are similar to cells at other stages, we conclude that they are probably not involved in production of vitellogenins. The observed decrease in reserve material after oviposition was caused by starvation due to lack of feeding during oogenesis and the moulting process. Evidence of energy reserve functions of storage cells during starvation periods (assumed also by Reuner et al. [15]) were observed in *Macrobiotus sapiens* Binda and Pilato, 1986 and other tardigrades, where storage cell size was found to be smaller after starvation [8, 9, 11, 14] and were also related to the stage of oogenesis [8, 12, 14, 46]. The size and content of reserve material in storage cells is also species dependent, e.g., three types of reserve material spheres were found in *H*. *exemplaris*, *M*. *polonicus* and *I*. *g*. *granulifer*, whereas only one type was found in *Xerobiotus pseudohufelandi* (Iharos, 1966) [14]. In active specimens of *R*. *coronifer*, we found large amounts of polysaccharides and lipids but low amount of proteins, similar to *X*. *pseudohufelandi* [14]. In *I*. *g*. *granulifer* large amounts of polysaccharides but fewer lipids and proteins were observed, and in *H*. *exemplaris* and *M*. *polonicus*, primarily lipids were observed [14]. In contrast, with these species that inhabit limnic habitats, both *R*. *coronifer* and *X*. *pseudohufelandi* inhabit dry terrestrial environments and are able to survive long periods of drought in the anhydrobiotic state [49, 50]. This supports the suggestion by Hyra et al. [14] that interspecies variability in storage cells is related to habitats and anhydrobiotic properties.

The storage cells of Type II were found in much smaller numbers (7.2% in all analyzed specimens) and only in females but with a similar ultrastructure during all oogenetic stages. These cells had few organelles and did not contain nucleolar vacuoles. In general, the youngest nucleoli are homogenous and do not possess nucleolar vacuoles [43], and it is possible that these storage cells represent young undifferentiated cells, perhaps stem cells. In general, stem cells are characterized as undifferentiated, unspecialized cells with simpler morphology compared to specialized cells from the same lineage [51]. Polymorphism of coelomocytes has also been verified in earthworms [52, 53], nematodes [54], echinoderms [55] and sea urchins [56]. The classification of coelomocytes is mostly based on differential staining, ultrastructure, and granule composition, as well as on behavioural traits (such as a tendency to form aggregations or filopodia in some cell types) but is still uniformly unsatisfactory, mostly due to various functional states and stages of maturation [57]. The classification of coelomocytes in earthworms is not well standardized, and the number and size of different coelomocytes can vary from species to species [52, 58]. However, it is assumed that coelomocyte types are derived from a common stock of stem cells, and different types of coelomocytes may be produced by direct transformation from stem cells [59]. Our study might be the first to ultrastructurally indicate the possible stem cells of tardigrade storage cells.

### Exposures to long-term desiccation and heating

The results of this study suggest that storage cells of the eutardigrade *R*. *coronifer* are not affected ultrastructurally by six months of desiccation or by heating at 50°C for 24 h. Still, heating of the tuns tended to considerably decrease survival of the animals. Additionally, the time of rehydration required to revive the animals tended to be longer for tuns exposed to heating. Thus, there were no indications that effects on viability of induced stress were connected with changes in the general structures of storage cells. Ramløv and Westh [25] did not find any effects on survival after heating *R*. *coronifer* for one hour at 50–70°C, while survival declined to approximately 20% at 80°C and to zero at 100°C. Since in our study the specimens were desiccated for six months before heating at 50°C, it is possible that this made them more vulnerable to heat stress. Since repair mechanisms are not working during anhydrobiosis, damage due to oxidative reactions with surrounding air accumulates over time [26]. Even if the non-heated animals did not express reduced survival after the six-month period, it may have made the body more vulnerable to damage by heat or unable to repair the inclusive damage from long-term desiccation plus heating. These detrimental effects apparently did not arise from damage to general cell structures but rather to molecular components necessary for cell survival. Protein denaturation occurs in cellular organelles during heat shock at temperatures of 42-45°C [60, 61, 62], and sub-lethal heat shock may also inactivate transcription, splicing and translation of mRNAs into proteins and alters cell morphology [60].

Relative humidity is another factor that may affect survival in desiccated tardigrades exposed to heat. Ramløv and Westh [25] suggested that the relative humidity at which animals were kept before heating (even at RH levels as low as 50%) may cause damage to cell components such as proteins (denaturation) when exposed to high temperatures through residual water present in the tun. In the eutardigrade *Paramacrobiotus richtersi* (Murray, 1911), very low humidity (0–3% RH) resulted in significantly higher survival after continuous exposure to 37°C for up to 21 days. Animals desiccated within their natural substrate (leaf litter) seemed to be less sensitive than animals desiccated and kept on blotted paper and did not show reduced survival when kept at 30–40% RH [26]. In our study, specimens were desiccated at 95% RH but were then kept in plastic bags under ambient laboratory conditions (room temperature, RH not monitored) until the heating exposure.

Vitrification has been proposed as a mechanism for survival in the anhydrobiotic state, whereby membranes and other cell components are stabilized in the absence of water in a non-crystalline amorphous solid (“glassy”) state that prevents cellular damage [63]. Evidence for the vitrification hypothesis was reported for the Macrobiotidae family within tardigrades [22], to which *R*. *coronifer* belongs. Although our observations of few differences in cell structures between hydrated and desiccated animals are in line with the prediction of the vitrification hypothesis that cells are “frozen” in a glassy state, they do not provide direct support for it.

We detected large amounts of lipids and polysaccharides but low amounts of protein in the storage cell cytoplasm of all examined specimens. Lipids have been proposed to have a key role in heat stress management of cells [64], but in anhydrobiotic processes, their role remains unclear. For anhydrobiotic nematodes, some have suggested that lipid reserves are not directly involved in processes of anhydrobiosis [65], whereas others have suggested a direct relationship between lipids/carbohydrates and successful anhydrobiosis [66]. Kinchin [46] proposed that different animal groups may have different mechanisms and that in tardigrades, lipids might be utilized during anhydrobiosis by conversion to glycerol or trehalose, which may stabilize membrane and protein structures [27]. Lipids might also serve as an energy source for metabolic preparations during anhydrobiotic induction or be used for energy after rehydration [67]. More studies on the role of lipids in storage cell physiology and anhydrobiosis would be valuable.

In conclusion, in our study we found (1) two types of storage cells in females of *R*. *coronifer*, while only one type in one male studied; (2) the ultrastructure of the storage cells of the first type changes during the process of oogenesis, while the ultrastructure of the second type of cells does not change; (3) that cells of the second type possibly represent stem cells for storage cells; (4) that storage cells (heated and non-heated specimens) accumulated large amount of lipids and polysaccharides, whereas the amount of proteins is low; (5) that exposure to 24 h of heating at 50°C following six months of desiccation reduced animal survival to 40%, while all non-heated animals recovered; and (6) no large differences in the ultrastructure of the storage cells between heated and non-heated desiccated specimens.

## Supporting information

S1 Table The average diameter of storage cells in active and dehydrated animals. Estimates represent individual averages based on measurements of 50 cells per animal.
(DOC)

S1 File Storage cells diameter (μm). Measurements of storage cells diameter in 50 active and 50 dehydrated specimens.
(XLS)
