# Supplementary material for: A comparative ultrastructure study of storage cells in the eutardigrade Richtersius coronifer in the hydrated state and after desiccation and heating stress
Source: PLoS One. 2018 Aug 10;13(8):e0201430. doi: 10.1371/journal.pone.0201430 (PMC6086413; doi:10.1371/journal.pone.0201430)
Supplement: S1 Table — Estimates represent individual averages based on measurements of 50 cells per animal. (DOC) [file pone.0201430.s001.doc]

**S1 Table. The average diameter of storage cells in active and dehydrated animals.** Estimates represent individual averages based on measurements of 50 cells per animal.

|  | **ACTIVE STATE**  [Hydrated animals] | **TUN STATE**  [Dehydrated animals] |
| --- | --- | --- |
| Number of individuals | 5 | 5 |
| Sum of measured cells  (50 storage cells per individual) | 250 | 250 |
| Range (Min–Max) of averages [µm] | 0.34 (15.27 – 15.61) | 0 |
| Range (Min–Max) of all cells [µm] | 9.55 (11.58 – 21.13) | 6.86 (8.45 – 15.31) |
| Average diameter ± SD [µm] | 15.36 ± 0.14 | 11.80 ± 0 |
